# Privacy-Preserving Authentication Protocol for Wireless Body Area Networks in Healthcare Applications

**DOI:** 10.3390/healthcare9091114

**Published:** 2021-08-28

**Authors:** Hyunho Ryu, Hyunsung Kim

**Affiliations:** School of Computer Science, Kyungil University, Gyeongsan-si 38428, Korea; ryoofamily0430@gmail.com

**Keywords:** healthcare service, body area network, privacy, authentication, security protocol

## Abstract

Mobile healthcare service has become increasingly popular thanks to the significant advances in the wireless body area networks (WBANs). It helps medical professionals to collect patient’s healthcare data remotely and provides remote medical diagnosis. Since the health data are privacy-related, they should provide services with privacy-preserving, which should consider security and privacy at the same time. Recently, some lightweight patient healthcare authentication protocols were proposed for WBANs. However, we observed that they are vulnerable to tracing attacks because the patient uses the same identifier in each session, which could leak privacy-related information on the patient. To defeat the weakness, this paper proposes a privacy-preserving authentication protocol for WBANs in healthcare service. The proposed protocol is only based on one-way hash function and with exclusive-or operation, which are lightweight operations than asymmetric cryptosystem operations. We performed two rigorous formal security proofs based on BAN logic and ProVerif tool. Furthermore, comparison results with the relevant protocols show that the proposed protocol achieves more privacy and security features than the other protocols and has suitable efficiency in computational and communicational concerns.

## 1. Introduction

Advances in mobile networking for Internet of Things (IoT) are powering the fourth industrial revolution. It connects physical things with digital worlds and allows for better collaboration and access across network participants, application services and people [1,2,3,4,5]. Wireless sensor network (WSN) technology is an essential component of IoT because it consists of a collection of sensors connected wirelessly. In the diverse kinds of WSNs, wireless body area network (WBAN) is a highly suitable communication network for medical IoT devices [6,7,8,9]. Healthcare services based on WBAN could provide remote mechanisms to monitor and collect patient’s health data. The distance between patients and professional doctor can affect health status [10,11,12,13]. However, locational inequality in the medical system such as lower hospital and professional doctor is a problem that exists in almost all countries [14,15]. However, the remote healthcare system can be helpful for this problem. Especially, the remote healthcare system is beneficial for chronic diseases such as diabetes, heart failure, and chronic obstructive pulmonary disease [16]. And chronic diseases are an increasingly important concern for remote healthcare systems [17]. Because the remote healthcare system can check a patient’s health status anytime and anywhere. In addition, since the patient’s health status is checked in real-time, it has the advantage of able to cope quickly and the doctor can early diagnosis if the patient’s health status become emergency [18,19]. Additionally, remote healthcare monitoring allows people to continue to stay at home rather than in expensive healthcare facilities such as hospitals or nursing homes [20,21].

However, privacy and security play key roles in protecting these data during data collection and transmission since remote healthcare service is vulnerable to various attacks [22,23,24,25,26,27,28,29]. If any attacker successfully launches the attacks, unintended functions may be performed via WBAN and these can cause a life threat to the patient. Therefore, it is imperative to devise authentication and key establishment protocols for securing remote healthcare applications.

There have been many authentication protocols for WBANs in healthcare applications [30,31,32,33,34,35,36,37,38,39,40,41]. Especially, the first anonymous authentication protocol based on smartcards was proposed by Zhu et al., which provides authentication with one round message communication but keeps user anonymity [30]. However, Lee et al. showed that Zhu et al.’s protocol cannot provide perfect user anonymity and backward secrecy and proposed an enhanced protocol [31]. Zhu et al.’s protocol and Lee et al.’s protocol were based on hash operations, a symmetric key cryptography and exclusive-or operations. Memon et al. proposed an anonymous authentication protocol for location-based services, which is based on elliptic curve cryptography (ECC) [32]. Soon after Reddy et al. showed vulnerabilities of Memon et al.’s protocol focused on key compromised impersonation attack, insider attack and insecure password changing phase and a problem of imperfect mutual authentication. Reddy et al. also proposed a two-factor authentication protocol based on ECC and smartcards [33]. Memon et al.’s protocol and Reddy et al.’s protocol are depending on asymmetric key cryptography, especially ECC. For the telecare medicine information system, Khatoon et al. and Ostad-Sharif et al. separately proposed authentication and key agreement protocol based on ECC [34,35]. By adopting a fuzzy extractor for the identification of patients using biometrics, Khatoon et al.’s protocol purposed to provide secure and privacy-preserving of the patient, bilinear pairing-based, unlinkable, mutual authentication and key agreement [34]. Ostad-Sharif et al. designed an anonymous and unlinkable authentication and key agreement protocol to provide perfect forward secrecy, which provided the formal security analysis using simulation tool AVISPA result [35]. Apart from the research efforts, Ali et al. proposed an authentication and access control protocol for securing wireless healthcare WSNs [36]. Ali et al.’s protocol is based on ECC and bilinear pairing and is proven to be secure based on AVISPA tool and Burrows–Abadi–Needham (BAN) logic [37].

Primitives based on ECC or bilinear pairing have computational overhead than any other cryptographic primitives and thereby they are heavily weighted on WBANs. To cope with the overhead, Khan et al. proposed an anonymous biometric-based authentication protocol using chaotic maps [38]. To use biometrics in the protocol, Khan et al. hired the Chebyshev chaotic map and hash function, which is a lightweight authentication cryptographic primitives. Aman et al. proposed a lightweight authentication protocol over WBANs, which are based on physical unclonable functions (PUFs) [39]. Aman et al.’s protocol is based on hash functions and exclusive-or operations. Even if two protocols from Khan et al. and Aman et al. provide operational efficiency, PUF assumption is a big burden to WBANs environment. Xu et al. proposed a lightweight anonymous authentication and key agreement protocol for WBANs without using the chaotic map nor PUFs [40]. Their protocol is only based on a hash function and exclusive-or operations and has an advantage in operational cost. However, Alzahrani et al. showed that Xu et al.’s protocol is vulnerable against replay attacks and key compromise impersonation attacks and suffers from the offline identity-guessing attack [41]. Furthermore, they proposed an improved protocol for WBANs in healthcare applications. Even though Alzahrani et al.’s protocol provides a lightweight computational overhead with various advantages on security and privacy concerns, we found that Alzahrani et al.’s protocol does not provide unlinkability of patients because it uses the same identifier of access point in each session.

The contributions of this paper are as follows:

(1) A new privacy-preserving authentication protocol for WBANs in remote healthcare applications is devised. In the protocol, an entity could protect privacy and security with a session key establishment for secure communication.

(2) The proposed protocol utilizes lightweight operations, which are based on the hash function and exclusive-or operation. This makes the protocol suitable for WBANs in remote healthcare applications.

(3) The formal security proof in BAN logic [37] demonstrates that the proposed protocol supports privacy and security. The formal security verification with ProVerif tool [42] shows that the proposed protocol can withstand both passive and active attacks. The informal analysis of its privacy and security is presented to verify the robustness of the proposed protocol against the well-known attacks.

(4) Efficiency analysis is done based on the complexity analysis of computation and communication overheads. The results show that the proposed protocol has a little overhead than the existing protocols.

The remainder of this paper is structured as follows. Section 2 summarizes the preliminaries of the research focused on healthcare system configuration, CK threat model and design goals. Section 3 gives a detailed description of the proposed privacy-preserving authentication protocol for remote healthcare applications. Section 4 demonstrates the formal, semi-formal and informal privacy and security results of the proposed protocol. Section 5 shows performance results focused on computation and communication. Section 6 provides discussion of importance of this research with future works. Section 7 concludes the work.

## 2. Preliminaries

In the digital age, hospitals and health service providers have pursued innovations for rich healthcare services. WBAN technology allows patients to be treated always even in remote areas and enables doctors to diagnose diseases and treat patients in medical institutions. And its technology can help anyone to easily access medical information [43]. It also serves to reduce patient anxiety by providing easy access to current medical information such as coronavirus disease 2019 (COVID-19). This section briefly reviews a system configuration for the target remote healthcare service and the design goals of the proposed protocol.

### 2.1. System Configuration

**Figure 1:**
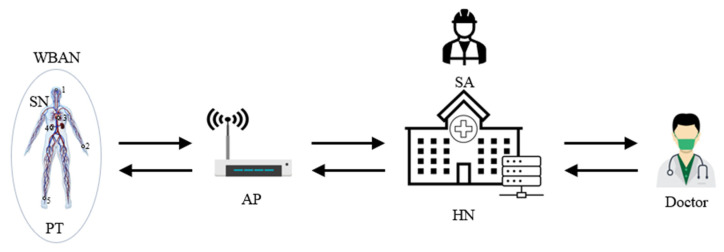
System configuration for remote healthcare service.

The target remote healthcare service is based on WBAN for patients. As shown in Figure 1, there are three main entities, which are a patient (PT) with some sensor nodes (SNs) on WBAN, access point (AP) and hub node (HN) as a server of the remote healthcare system. Especially, a system administrator (SA) is required for the system set-up but HN could do this role instead if it is necessary. The roles of each entity are defined as follows:

- SA: It sets up system parameters and registers participants by deploying important secret values in the memory of each party.
- HN: It has a very important role as the central server for the healthcare service, which collects and keeps a database of electronic health records (EHRs) for the registered PTs. In addition to this, it works also as a registration center for all network participants and issues SNs and APs for PTs. Furthermore, it works as an authentication server to check the authenticity of system entities.
- AP: AP works as a communication gateway from SN to HN and vice versa via wireless communication link. Thereby, it does not perform any validation of messages. It is assumed that an AP belongs to a specific PT only.
- SN: Some SNs are deployed on a PT, as notated as 1, 2, 3, 4 and 5 in the left part of Figure 1, to form a WBAN by HN or SA, which do the role of collecting EHR data of the PT and transmitting them to HN. An SN has sensors to check the purposed health status such as body temperature, blood pressure, electrocardiogram and so on. It needs to consider EHR privacy because the healthcare service is data sensitive.
- PT: PT is a subject of the remote healthcare service. Normally, PT does not take part in the network communication directly but subscribes the service to SA or HN. Then, SA or HN issues some SNs and an AP of the PT for the service.
- Doctor: Doctors make the diagnosis based on PT’s EHRs by accessing HN. Doctors need to regularly check the health status of PTs and provide proper medical treatments via on-line.

### 2.2. CK Threat Model

This subsection describes the widely accepted and well-known Canetti and Krawczyk (CK) threat model, which defines the ability of an adversary and is one of the foundations for formal privacy and security analysis on cryptographic protocol [44,45]. In the CK model, the adversary can fully control the communication links by listening to, altering, deciding on and injecting into the transferring information. Apart from these basic adversarial capabilities, in this model, it is assumed that the adversary can obtain secret information stored in the parties’ memories via explicit attacks. As a result, the security of an authentication protocol should guarantee that the leakage of private values, such as session ephemeral secrets, would have the least possible influence on the security of other sessions and other private credentials of the communicating entities.

### 2.3. Design Goals

The healthcare system should provide privacy and security at the same time [46,47]. Normally, only anonymity is considered to provide privacy of PT in some other protocols in [40,41]. However, we also need to further consider unlinkability as another important privacy feature. To design a new authentication protocol for the remote healthcare service based on the CK threat model, the following five security properties and two privacy requirements are considered in this paper.

[SP1] Mutual authentication: To allow only authorized PT to get the medical services provided by HN, mutual authentication between SN and HN is required.

[SP2] Session key agreement: After a successful process of mutual authentication, further EHR data communications between SN and HN should be encrypted based on the session key to achieve confidentiality and integrity.

[SP3] Message freshness: Each entity in the system needs to check message freshness to cope with various attacks. It could be supported either by using timestamp or random nonce.

[SP4] Perfect forward secrecy: It could assure that the security of the system will not be compromised even if long-term secrets used in the protocol are compromised.

[SP5] Attack resistance: Due to the open environment in the remote healthcare service, the transmitted messages among network entities may be intercepted, modified and replayed by the adversary. Therefore, the proposed authentication protocol should be able to withstand various attacks, such as replay attack, impersonation attack, man-in-the-middle attack and known session-specific temporary information attack.

[PP1] Anonymity: Anonymity is an important privacy feature in the remote healthcare service. To protect the identity privacy of PT, the proposed protocol should guarantee that no one can get the PT’s identity from the intercepted messages on the public channels.

[PP2] Unlinkability: Unlinkability is another important privacy feature in the remote healthcare service, which guarantees that the adversary cannot distinguish whether these different session’s messages are related or not. The cryptographic protocol should not only guarantee the PT’s anonymity but also provide unlinkability between sessions.

## 3. Proposed Authentication Protocol

In this section, a privacy-preserving authentication protocol for WBANs in healthcare service is proposed. The proposed protocol uses only the hash function with exclusive-or operations to provide the design goals. We assume that all the participants are synchronized on time using any proper scheme and a maximum transmission delay ∆*t* is agreed on mutually. The proposed protocol consists of four phases, i.e., initialization phase, registration phase, authentication phase and identity modification phase. First of all, the initialization phase sets up a security building block for the overall network. PT possessed with SN and AP is a target for the registration phase to either SA or HN. The authentication phase is for the basic security service to check whether the entity is legal or not and is also to set up a session key for further secure communications. The identity modification phase is used when PT wants to change SN’s identity for privacy reasons. Table 1 defines the symbols and their meanings used in this paper.

### 3.1. Initialization Phase

For the system initialization, SA performs the following steps.

Step 1. SA selects a long-term master key *KS_HN_* for HN.
Step 2. SA stores *KS_HN_* in the memory of HN.

### 3.2. Registration Phase

**Figure 2:**
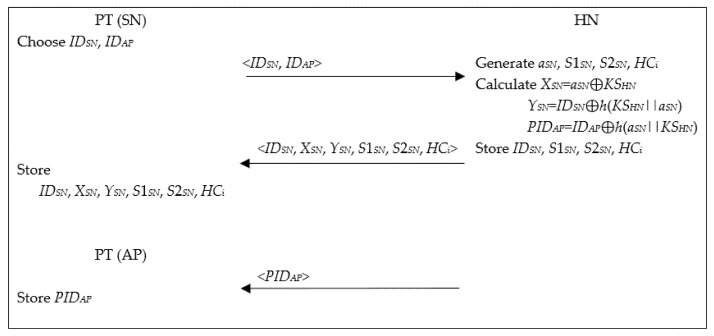
Registration phase.

When a PT wants to subscribe to a remote healthcare service, HN performs the following steps after issuing SN and AP for PT as shown in Figure 2. All parameters are established by HN for WBANs over a secure channel.

Step 1. PT chooses two identities *ID_SN_* and *ID_AP_* for SN and AP, respectively, and sends them to HN. After receiving the information, HN generates four random numbers *a_SN_*, *S*1*_SN_*, *S*2*_SN_* and *HC_i_* for SN, forms a set <*ID_SN_*, *S*1*_SN_*, *S*2*_SN_*, *HC_i_*> and stores it in the memory.
Step 2. After that, HN calculates *X_SN_* = *a_SN_* ⊕ *KS_HN_*, *Y_SN_* = *ID_SN_* ⊕ *h*(*KS_HN_*||*a_SN_*) and *PID_AP_* = *ID_AP_* ⊕ *h*(*a_SN_*||*KS_HN_*), composes a set <*ID_SN_*, *X_SN_*, *Y_SN_*, *S*1*_SN_*, *S*2*_SN_*, *HC_i_*> and stores it in the memory of SN. They are used for authenticity check of PT.
Step 3. HN stores *PID_AP_* in the memory of AP.

### 3.3. Authentication Phase

**Figure 3:**
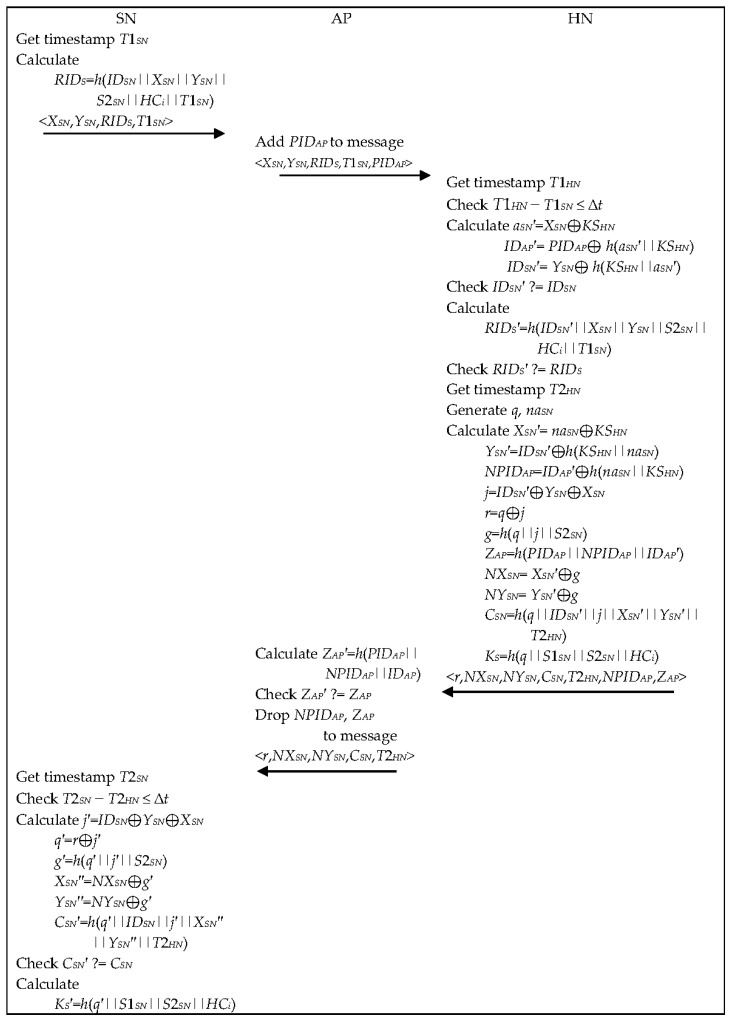
Authentication phase.

When a PT wants to use the subscribed remote healthcare service, PT with SN and AP needs to use this phase to log-in HN as shown in Figure 3. SN does whole roles of PT periodically to send the predefined sensed EHR data to HN via AP. This phase has two purposes, mutual authentication and session key agreement. Timestamp in each message is used to provide message freshness, which is used to cope with the replay attack. The detailed steps are as follows:

Step 1. SN gets the current timestamp *T*1*_SN_*, calculates a message authentication code *RID_S_* = (*ID_SN_*||*X_SN_*||*Y_SN_*||*S*2*_SN_*||*HC_i_*||*T*1*_SN_*) and composes a message {*X_SN_*, *Y_SN_*, *RID_S_*, *T*1*_SN_*} to submit to AP.
Step 2. When AP receives the message, it adds a session dependent pseudo identity *PID_AP_* to the message {*X_SN_*, *Y_SN_*, *RID_S_*, *T*1*_SN_*, *PID_AP_*} and sends the message to HN.
Step 3. When HN receives the message, it gets the current timestamp *T*1*_HN_* and verifies the freshness of the message by validating *T*1*_HN_* − *T*1*_SN_* ≤ ∆*t* where ∆*t* is the allowed transmission delay of the network. If it does not hold, HN treats this message as a replay attack and aborts the session. Otherwise, HN calculates *a_SN_*′ = *X_SN_* ⊕ *KS_HN_* and *ID_AP_*′ = *PID_AP_* ⊕ *h*(*a_SN_*′||*KS_HN_*). After that, HN calculates *ID_SN_*′ = *Y_SN_* ⊕ *h*(*KS_HN_*||*a_SN_*′) and compares it with *ID_SN_* stored in its memory. Only if the verification is successful, HN calculates *RID_S_*′ = *h*(*ID_SN_*′||*X_SN_*||*Y_SN_*||*S*2*_SN_*||*HC_i_*||*T*1*_SN_*) using the parameters in its repository. Finally, HN checks whether *RID_S_*′ is equal to *RID_S_* or not.
Step 4. Only after all verifications are successful, HN could believe the authenticity of SN and AP and forms a reply message with two options, one is to be authenticated to SN and AP and another is to update the authentication parameters for the next authentication for SN and AP, respectively. For this, HN gets the current timestamp *T*2*_HN_*, generates two random numbers *q* and *na_SN_*, and calculates *X_SN_*′ = *na_SN_* ⊕ *KS_HN_*, *Y_SN_*′ = *ID_SN_*′ ⊕ *h*(*KS_HN_*||*na_SN_*), *NPID_AP_* = *ID_AP_*′ ⊕ *h*(*na_SN_*||*KS_HN_*), *j* = *ID_SN_*′ ⊕ *Y_SN_* ⊕ *X_SN_*, *r* = *q* ⊕ *j*, *g* = *h*(*q*||*j*||*S*2*_SN_*), *Z_AP_* = *h*(*PID_AP_*||*NPID_AP_*||*ID_AP_*′), *NX_SN_* = *X_SN_*′ ⊕ *g*, *NY_SN_* = *Y_SN_*′ ⊕ *g*, *C_SN_* = *h*(*q*||*ID_SN_*′||*j*||*X_SN_*′||*Y_SN_*′||*T*2*_HN_*) and *K_S_* = *h*(*q*||*S*1*_SN_*||*S*2*_SN_*||*HC_i_*). After that, HN overwrites *S*1*_SN_* into *S*2*_SN_* and changes *S*2*_SN_* with *K_S_* in its memory, which are used for the next authentication for privacy provision. And then, HN calculates *HC_i_*′ = *h*(*HC_i_*) and replaces it to *HC_i_* as *HC_i_* = *HC_i_*′, which is for updating the session key parameter. After that, HN composes a message {*r*, *NX_SN_*, *NY_SN_*, *C_SN_*, *T*2*_HN_*, *NPID_AP_*, *Z_AP_*} and sends it to AP.
Step 5. After receiving the message, AP checks the freshness of message by calculating *Z_AP_*′ = *h*(*PID_AP_*||*NPID_AP_*||*ID_AP_*) and verifying whether *Z_AP_*′ is the same as *Z_AP_* in the message or not. Only if the verification is successful, AP overwrites *NPID_AP_* into *PID_AP_* in its memory. After that, AP drops *NPID_AP_* and *Z_AP_* from the message and sends the reformed message {*r*, *NX_SN_*, *NY_SN_*, *C_SN_*, *T*2*_HN_*} to SN.
Step 6. When SN receives the message, it gets the current timestamp *T*2*_SN_* and verifies the freshness of the message by validating *T*2*_SN_* − *T*2*_HN_* ≤ ∆*t*. If it is not successful, SN aborts the session, which is treated as a replay attack. Otherwise, it calculates *j*′ = *ID_SN_* ⊕ *Y_SN_* ⊕ *X_SN_*, *q*′ = *r* ⊕ *j*′, *g*′ = *h*(*q*||*j*′||*S*2*_SN_*), *X_SN_*″ = *NX_SN_* ⊕ *g*′, *Y_SN_*″ = *NY_SN_* ⊕ *g*′ and *C_SN_*′ = *h*(*q*′||*ID_SN_*||*j*′||*X_SN_*″||*Y_SN_*″||*T*2*_HN_*) and validates *C_SN_*′ by comparing it with *C_SN_* in the message. It aborts the session if the validation fails. Otherwise, SN implicitly accept the authenticity of HN and calculates a session key *K_S_*′ = *h*(*q*′||*S*1*_SN_*||*S*2*_SN_*|| *HC_i_*) and overwrite *S*1*_SN_* into *S*2*_SN_* and changes *S*2*_SN_* with *K_S_*. SN replaces the two parameters, *X_SN_*″ and *Y_SN_*″ into *X_SN_* and *Y_SN_*, respectively, which are the next authentication parameters. Finally, SN calculates *HC_i_*′ = *h*(*HC_i_*) and replaces it to *HC_i_* as *HC_i_* = *HC_i_*′, which is for updating the session key parameter.

### 3.4. Identity Modification Phase

Whenever a PT wants to change his (or her) identity, this phase should be performed. To change identity of PT, SN sends the identity modification request to HN. Then HN provides identity modification parameter only after the successful authentication. The phase is performed as follows:

Step 1. SN sets the current timestamp *T*1*_SN_*, selects a new identity *ID_SN_^NEW^*, calculates *NID_SN_* = *ID_SN_^NEW^* ⊕ *S*2*_SN_* and *RID_S_* = *h*(*ID_SN_*||*X_SN_*||*Y_SN_*||*S*2*_SN_*||*NID_SN_*||*HC_i_*||*T*1*_SN_*), composes a message {*X_SN_*, *Y_SN_*, *RID_S_*, *T*1*_SN_*, *NID_SN_*} and submits it to AP.
Step 2. When AP receives the message, it adds *PID_AP_* to the message {*X_SN_*, *Y_SN_*, *RID_S_*, *T*1*_SN_*, *NID_SN_*, *PID_AP_*} and sends the message to HN.
Step 3. When HN receives the message, it sets the current timestamp *T*1*_HN_*. And HN validates the freshness of the message by verifying *T*1*_HN_* − *T*1*_SN_* ≤ ∆*t*. If *T_SN_* is not fresh, HN aborts the session. Otherwise, HN calculates authentication parameters *a_SN_*′ = *X_SN_* ⊕ *KS_HN_* and *ID_AP_*′ = *PID_AP_* ⊕ *h*(*a_SN_*′||*KS_HN_*). After that, HN calculates *ID_SN_*′ = *Y_SN_* ⊕ *h*(*KS_HN_*||*a_SN_*′) and compares it with *ID_SN_* stored in its memory. Only if the verification is successful, HN calculates *RID_S_*′ = *h*(*ID_SN_*′||*X_SN_*||*Y_SN_*||*S*2*_SN_*||*NID_SN_*|| *HC_i_*||*T*1*_SN_*) using the parameters in its repository. Finally, HN checks whether *RID_S_*′ is equal to *RID_S_*.
Step 4. Only after all verifications are successful, HN withdraws the new identity from SN by computing *ID_SN_^NEW^*′ = *NID_SN_* ⊕ *S*2*_SN_*. After that, HN generates current timestamp *T*2*_HN_* and random numbers *q* and calculates the new identity related authentication parameters *Y_SN_*′ = *ID_SN_^NEW^*′ ⊕ *h*(*KS_HN_||a_SN_*′), *j* = *ID_SN_* ⊕ *Y_SN_* ⊕ *X_SN_*, *r* = *q* ⊕ *j*, *g* = *h*(*q*||*j*||*S*2*_SN_*), *NY_SN_* = *Y_SN_*′ ⊕ *g* and *C_SN_* = *h*(*q*||*ID_SN_*||*j*||*Y_SN_*′||*T*2*_HN_*). Then HN overwrites *ID_SN_ ^NEW^*′ into *ID_SN_* in its memory. Next HN composes a message {*r*, *NY_SN_*, *C_SN_*, *T*2*_HN_*} and sends it to AP.
Step 5. After receiving the message, AP sends the message {*r*, *NY_SN_*, *C_SN_*, *T*2*_HN_*} to SN.
Step 6. When SN receives the message, it sets the current timestamp *T*2*_SN_*. And SN validates the freshness of the message by verifying *T*2*_SN_* − *T*2*_HN_* ≤ ∆*t*. If *T*2*_HN_* is not fresh, SN aborts the session. Otherwise, SN calculates *j*′ = *ID_SN_* ⊕ *Y_SN_* ⊕ *X_SN_*, *q*′ = *r* ⊕ *j*′, *g*′ = *h*(*q*||*j*′||*S*2*_SN_*), *Y_SN_*″ = *NY_SN_* ⊕ *g*′ and *C_SN_*′ = *h*(*q*′||*ID_SN_*||*j*′||*Y_SN_*″||*T*2*_HN_*), which are withdrawing the new identity related authentication parameters. After that, SN validates *C_SN_*′ by comparing it with *C_SN_* in the message. It aborts the session if the validation fails. Otherwise, SN replaces *Y_SN_* with *Y_SN_*″ in its memory.

## 4. Security and Privacy Results

This section provides security analysis of the proposed protocol by using BAN logic and ProVerif tool based on the CK threat model [37,42]. Then, we demonstrate that the proposed protocol can achieve higher privacy and security features than the other related protocols.

### 4.1. BAN Logic Result

In this subsection, we analyze the security of the proposed protocol based on BAN logic. BAN logic is a widely adopted major formal method of valuation of any authentication protocol. BAN logic analyses using axioms to verify message origin, message freshness and faithful of the origin of the message [37]. The notations in formal security analysis for BAN logic are listed as follows:

- *Q*|≡ *X*: Principal *Q* believes the statement *X*.
- #(*X*): Formula *X* is fresh.
- *Q*|⟹*X*: Principal *Q* has jurisdiction over the statement *X*.
- *Q*⊲*X*: Principal *Q* sees the statement *X*.
- *Q*|~*X*: Principal *Q* once said the statement *X*.
- (*X*, *Y*): Formula *X* or *Y* is one part of the formula (*X*, *Y*).
- ${\langle P\rangle }_{Q}$: Formula *P* combined with the formula *Q*.
- $Q\overset{SK}{↔}R$: Principal *Q* and *R* may use the shared session key, *SK* to communicate among each other. *SK* is good, in that any principal except *Q* and *R* will never discover it.

In addition, we use the following BAN logic rules to prove that the proposed protocol provides a secure mutual authentication between SN, AP and HN:

- Message-meaning rule: $\frac{R|\equiv R\overset{Y}{↔}S,\;R⊲{<X>}_{Y}}{R\left|\equiv S\right|\sim X}$
- Nonce-verification rule: $\frac{R\left|\equiv \;\#\left(X\right),\;R\right|\equiv S|\sim X}{R\left|\equiv S\right|\equiv X}$
- Jurisdiction rule: $\frac{R\left|\equiv S\right|⟹X,\;R\left|\equiv S\right|\equiv X}{R|\equiv X}$
- Freshness rule: $\frac{R|\equiv \;\#\left(X\right)}{R|\equiv \;\#\left(X,Y\right)}$

To show how the proposed protocol provide secure mutual authentication between SN and HN, we need to achieve the following goals:

**Goal 1:** HN|≡(HN$\overset{Ks}{↔}$SN**)**

**Goal 2:** SN|≡(SN$\overset{Ks}{↔}$HN**)**

**Goal 3:** HN|≡SN|≡(SN$\overset{Ks}{↔}$HN**)**

**Goal 4:** SN|≡HN|≡(HN$\overset{Ks}{↔}$SN**)**

**Idealized form:** The arrangement of the transmitted messages between *SN*, *AP* and *HN* in the proposed protocol to the idealized forms is as follows:

Message 1. SN → AP: <*X_SN_*>*_KS__HN_*, <*Y_SN_*>*_KS__HN_*, <*RIDs*>*_KS__HN_*, *T*1*_SN_*

Message 2. AP → HN: <*X_SN_*>*_KS_*_HN_, <*Y_SN_*>*_KS_*_HN_, <*RIDs*>*_KS_*_HN_, *T*1*_SN_*, <*PID_AP_*>*_KS__HN_*

Message 3. HN → AP: <*r*>*_KS__HN_*, <*NX_SN_*>*_KS__HN_*, <*NY_SN_*>*_KS__HN_*, <*C_SN_*>*_KS__HN_*, <*NPID_AP_*>*_KS__HN_*, <*Z_AP_*>*_KS__HN_*, *T*2*_HN_*

Message 4. AP → SN: <*r*>*_KS__HN_*, <*NX_SN_*>*_KS__HN_*, <*NY_SN_*>*_KS__HN_*, <*C_SN_*>*_KS__HN_*, *T*2*_HN_*

**Assumptions:** The following are the initial assumptions of the proposed protocol:

A1: HN|≡#(*T*1*_SN_*)

A2: HN|≡#(*T*2*_SN_*)

A3: SN|≡#(*T*1*_HN_*)

A4: SN|≡#(*T*2*_HN_*)

A5: SN|≡HN$\overset{X_{SN}}{↔}$SN

A6: HN|≡HN$\overset{X_{SN}}{↔}$SN

A7: SN|≡HN⟹HN$\overset{X_{SN}}{↔}$SN

A8: HN|≡SN⟹HN$\overset{X_{SN}}{↔}$SN

**Proof.**  In the following, we prove the test goals in order to show the secure authentication using BAN logic rules and the assumptions. □

Based on Message 1, we could derive:
 Step 1. AP⊲(<*X_SN_*>*_KS__HN_*, <*Y_SN_*>*_KS__HN_*, <*RIDs*>*_KS__HN_*, *T*1*_SN_*)
Based on Step 1, AP adds <*PID_AP_*>*_KS__HN_* to the message and sends it to HN. Based on Message 2, we could derive:
 Step 2. HN⊲(<*X_SN_*>*_KS__HN_*, <*Y_SN_*>*_KS__HN_*, <*RIDs*>*_KS__HN_*, *T*1*_SN_*, <*PID_AP_*>*_KS__HN_*)
According to assumption A6 and the message-meaning rule, we get:
 Step 3. HN|≡AP|~(<*X_SN_*>*_KS__HN_*, <*Y_SN_*>*_KS__HN_*, <*RIDs*>*_KS__HN_*, *T*1*_SN_*, <*PID_AP_*>*_KS__HN_*)
According to assumptions A1 and A2 and the freshness concatenation rule, we get:
 Step 4. HN|≡#(<*X_SN_*>*_KS__HN_*, <*Y_SN_*>*_KS__HN_*, <*RIDs*>*_KS__HN_*, *T*1*_SN_*, <*PID_AP_*>*_KS__HN_*)
According to Steps 3 and 4 and the nonce verification rule, we get:
 Step 5. HN|≡SN|≡(<*X_SN_*>*_KS__HN_*, <*Y_SN_*>*_KS__HN_*, <*RIDs*>*_KS__HN_*, *T*1*_SN_*, <*PID_AP_*>*_KS__HN_*)
According to Step 5, assumption A6 and the believe rule, we get:
 Step 6. HN|≡SN|≡(HN$\overset{KS_{HN}}{↔}$SN)
According to assumption A8 and the jurisdiction rule, we get:
 Step 7. HN|≡(HN$\overset{KS_{HN}}{↔}$SN)
According to Steps 5, 6 and 7 and the nonce verification rule, we conclude:
 Step 8. HN|≡SN|≡(SN$\overset{Ks}{↔}$HN) **(Goal 3)**
According to assumption A8 and the jurisdiction rule, we get:
 Step 9. HN|≡(HN$\overset{Ks}{↔}$SN) **(Goal 1)**
Based on Message 3, we could derive:
 Step 10. AP⊲(<*r*>*_KS__HN_*, <*NX_SN_*>*_KS__HN_*, <*NY_SN_*>*_KS__HN_*, <*C_SN_*>*_KS__HN_*, <*NPID_AP_*>*_KS__HN_*, <*Z_AP_*>*_KS__HN_*, *T*2*_HN_*)
According to the message meaning rule, we get:
 Step 11. AP|≡HN|~(<*r*>*_KS__HN_*, <*NX_SN_*>*_KS__HN_*, <*NY_SN_*>*_KS__HN_*, <*C_SN_*>*_KS__HN_*, <*NPID_AP_*>*_KS__HN_*, <*Z_AP_*>*_KS__HN_*, *T*2*_HN_*)
Based on Step 10, AP drops <*NPID_AP_*>*_KS__HN_* and <*Z_AP_*>*_KS__HN_* to the message and sends it to HN.
Based on Message 4, we derive:
 Step 12. SN⊲(<*r*>*_KS__HN_*, <*NX_SN_*>*_KS__HN_*, <*NY_SN_*>*_KS__HN_*, <*C_SN_*>*_KS__HN_*, *T*2*_HN_*)
According to assumption A5 and the message-meaning rule, we get:
 Step 13. SN|≡AP|~(<*r*>*_KS__HN_*, <*NX_SN_*>*_KS__HN_*, <*NY_SN_*>*_KS__HN_*, <*C_SN_*>*_KS__HN_*, *T*2*_HN_*)
According to assumptions A3 and A4 and the freshness concatenation rule, we get:
 Step 14. SN|≡#(<*r*>*_KS__HN_*, <*NX_SN_*>*_KS__HN_*, <*NY_SN_*>*_KS__HN_*, <*C_SN_*>*_KS__HN_*, *T*2*_HN_*)
According to Steps 12 and 13 and the nonce verification rule, we get:
 Step 15. SN|≡HN|≡(<*r*>*_KS__HN_*, <*NX_SN_*>*_KS__HN_*, <*NY_SN_*>*_KS__HN_*, <*C_SN_*>*_KS__HN_*, *T*2*_HN_*)
According to Step 14, assumption A5 and the believe rule, we get:
 Step 16. SN|≡HN|≡(HN$\overset{KS_{HN}}{↔}$SN)
According to assumption A7 and the jurisdiction rule, we get:
 Step 17. SN|≡(HN$\overset{KS_{HN}}{↔}$SN)
According to Steps 14, 15 and 16 and the nonce verification rule, we get:
 Step 18. SN|≡HN|≡(HN$\overset{Ks}{↔}S$N) **(Goal 4)**
According to assumption A7 and the jurisdiction rule, we get:
 Step 19. SN|≡(SN$\overset{Ks}{↔}$HN) **(Goal 2)**

According to Steps 9 and 19, the proposed authentication protocol successfully achieves the four goals. Both SN and HN could believe that they share the common session key *K_S_* = *K_S_*′ = *h*(*q*′||*S*1*_SN_*||*S*2*_SN_*).

### 4.2. ProVerif Result

ProVerif is an automated tool for verifying security in cryptographic protocol [42]. It is supposed to be based on the CK threat model for security verification. ProVerif is a powerful tool that can verify all the possible attacks regarding mutual authentication. It also can prove safety of security properties for mutual authentication. For ProVerif analysis, we first define two channels ch1 and ch2 as public channels, among SN, AP and HN. In the ProVerif analysis, we used svalueA and svalueB to validate the session dependency. There are four events to check mutual authentication between SN and HN, which are SHbegin(entity), HSbegin(entity), SHend(entity) and HSend(entity). Session key security could be proved based on two queries, query attacker(svalueA) and query attacker(svalueB) based on the shared session key. For the basic operations, we defined Hash(bitstring) and XOR(bitstring, bitstring) for a one-way hash function and an exclusive-or operation, respectively. After defining processes of each entity, we performed a ProVerif demo for the entities of SN, AP and HN.

We have configured the ProVerif code as follows:

  (*--The two public channel--*)

free ch1: channel.

free ch2: channel.

  (*--The basic type--*)

type entity.

type nonce.

type key.

  (*--Hash operation--*)

fun Hash(bitstring): bitstring.

  (*--XOR operation--*)

fun XOR(bitstring, bitstring): bitstring.

equation forall x: bitstring, y: bitstring;

XOR(XOR(x, y), y) = x.

  (*--Concat operation--*)

fun Con(bitstring, bitstring): bitstring.

fun Enc(bitstring,key): bitstring.

reduc forall x: bitstring, y: key;

Dec(Enc(x,y),y) = x.

  (*--Type convertion--*)

fun nontobit(nonce): bitstring [data,typeConverter].

fun bittokey(bitstring): key [data,typeConverter].

  (*--The basic variables--*)

free SN, AP, HN: entity. (*---three entities in the proposed protocol--*)

free T1SN: bitstring.

free T2HN: bitstring.

free S1SN: bitstring.

free S2SN: bitstring.

free HCi: bitstring.

free KSHN: bitstring[private]. (*---public key--*)

  (*--Authentication queries--*)

event SHbegin(entity).

event SHend(entity).

event HSbegin(entity).

event HSend(entity).

query t: entity; inj-event(SHend(t)) ==> inj-event(SHbegin(t)).

query t: entity; inj-event(HSend(t)) ==> inj-event(HSbegin(t)).

  (*--Queries--*)

free svalueA, svalueB: bitstring [private].

query attacker(svalueA);

attacker(svalueB).

  (*--SN--*)

let processSN(IDSN: bitstring, XSN: bitstring, YSN: bitstring) =

let (RIDs: bitstring) = Hash(Con(IDSN, Con(XSN, Con(YSN, Con(S2SN,

Con(HCi,T1SN)))))) in

event HSbegin(HN);

  (*-- SN > AP --*)

out(ch1, (XSN, YSN, RIDs, T1SN, true));

  (*-- AP > SN --*)

in(ch1, (r: bitstring, NXSN: bitstring, NYSN: bitstring, CSN: bitstring));

let (xj: bitstring) = XOR(IDSN, XOR(YSN, XSN)) in

let (xq: bitstring) = XOR(r, xj) in

let (xg: bitstring) = Hash(Con(xq, Con(xj, S2SN))) in

let (xXSN: bitstring) = XOR(NXSN, xg) in

let (xYSN: bitstring) = XOR(NYSN, xg) in

let (xCSN: bitstring) = Hash(Con(xq, Con(IDSN, Con(xj, Con(xXSN, Con(xYSN, T2HN)))))) in

if xCSN = CSN then

let (xKs: bitstring) = Hash(Con(xq, Con(S1SN, Con(S2SN, HCi)))) in

event SHend(SN);

out(ch1, Enc(svalueA, bittokey(xKs))).

  (*--AP--*)

let processAP(IDAP: bitstring, PIDAP: bitstring) =

in(ch1, (XSN: bitstring, YSN: bitstring, RIDs: bitstring));

  (*-- AP > HN --*)

out(ch2, (XSN, YSN, RIDs, T1SN, PIDAP, true));

  (*-- HN > AP --*)

in(ch2, (r: bitstring, NXSN: bitstring, NYSN: bitstring, CSN: bitstring, NPIDAP: bitstring, ZAP: bitstring));

let (xZAP: bitstring) = Hash(Con(PIDAP, Con(NPIDAP, IDAP))) in

if xZAP = ZAP then

  (*-- AP > SN --*)

out(ch1, (r, NXSN, NYSN, CSN, T2HN, true)).

  (*--HN--*)

let processHN(IDAP: bitstring, IDSN: bitstring) =

in(ch2, (XSN: bitstring, YSN: bitstring, RIDs: bitstring, PIDAP: bitstring));

let (a: bitstring) = XOR(XSN, KSHN) in

let (xIDAP: bitstring) = XOR(PIDAP,Hash(Con(a,KSHN))) in

let (xIDSN: bitstring) = XOR(YSN,Hash(Con(KSHN,a))) in

if xIDSN = IDSN then

let (xRIDs: bitstring) = Hash(Con(IDSN,Con(XSN,Con(YSN,Con(S2SN, Con(HCi, T1SN)))))) in

if xRIDs = RIDs then

event SHbegin(SN);

new q: nonce;

new nasn: nonce;

let (xXSN: bitstring) = XOR(nontobit(nasn),KSHN) in

let (xYSN: bitstring) = XOR(IDSN,Hash(Con(KSHN,nontobit(nasn)))) in

let (NPIDAP: bitstring) = XOR(IDAP,Hash(Con(nontobit(nasn),KSHN))) in

let (j: bitstring) = XOR(IDSN,XOR(YSN,XSN)) in

let (r: bitstring) = XOR(nontobit(q),j) in

let (g: bitstring) = Hash(Con(nontobit(q),Con(j,S2SN))) in

let (ZAP: bitstring) = Hash(Con(PIDAP,Con(NPIDAP,IDAP))) in

let (NXSN: bitstring) = XOR(xXSN,g) in

let (NYSN: bitstring) = XOR(xYSN,g) in

let (CSN: bitstring) = Hash(Con(nontobit(q), Con(IDSN, Con(j, Con(xXSN, Con(xYSN, T2HN)))))) in

let (Ks: bitstring) = Hash(Con(nontobit(q),Con(S1SN, Con(S2SN, HCi)))) in

  (*-- HN > AP --*)

out(ch2, (r, NXSN, NYSN, CSN, T2HN, NPIDAP, ZAP, true));

event HSend(HN);

out(ch2, Enc(svalueB, bittokey(Ks))).

  (*--Start process--*)

process(

new XSN: bitstring;

new YSN: bitstring;

new PIDAP: bitstring;

new IDSN: bitstring;

new IDAP: bitstring;

(!processSN(IDSN, XSN, YSN)) |

(!processAP(IDAP, PIDAP)) |

(!processHN(IDAP, IDSN))

)

**Figure 4:**
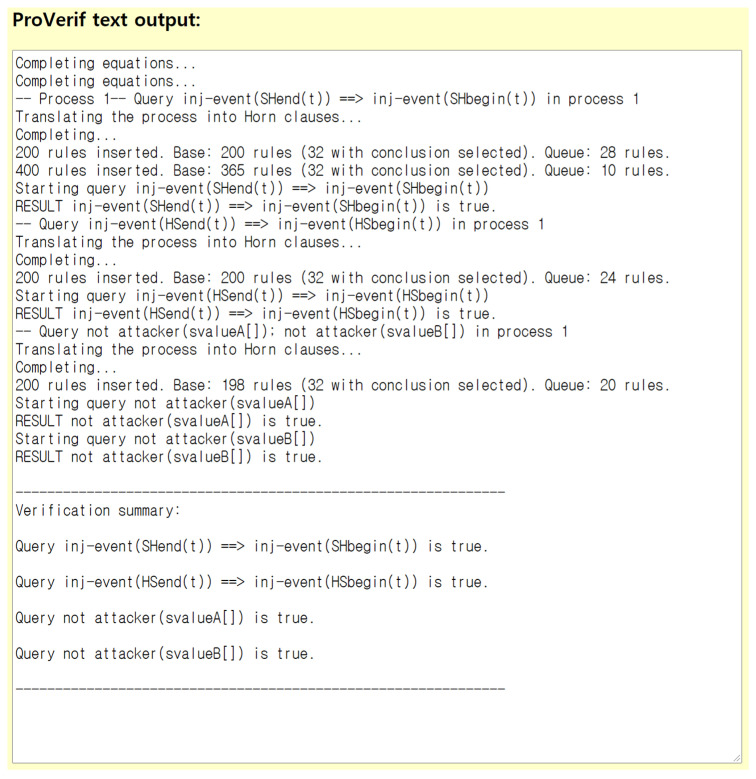
ProVerif result.

Figure 4 shows ProVerif result, which provides the successful security validation of the proposed protocol. From the result, we could find that “Query inj-event(SHend(t)) ==> inj-event(SHbegin(t)) is true.” and “Query inj-event(HSend(t)) ==> inj-event(HSbegin(t)) is true.” Those are to show mutual authentication property and replay attack resistance of the proposed protocol. After “Query not attacker (svalueA[]) is true.” and “Query not attacker (svalueB[]) is true.” show the anonymity of network participants and secrecy of the shared session key. It shows that the proposed protocol is properly performed by the tool without having any problems. As a result, we could conclude that the proposed protocol could establish a secure session key between SN and HN and the CK adversary could not discover the session key.

### 4.3. Informal Privacy and Security Analysis

As mentioned in [48], past research over the last thirty decades has told us that, a security proof is highly prone to be fallacious due to the adoption of an insufficient security model which fails to capture all the realistic capabilities of the adversary or due to a flawed or non-tight security reduction, and the field of provable security is a much an art as a science. While formal methods are often misused and reductionist security proofs are usually very intricate, turgid and prone to errors, particular care shall be given when conducting proof for an authentication protocol. To cope with the formal methods problems, this subsection is dedicated to present informal privacy and security analysis of the proposed protocol, which is focused on the privacy and security goals depicted in Section 2.3. For the CK threat model, we use the definition mentioned in Section 2.2. Table 2 shows the feature comparisons among the related protocols devised by Khatoon et al. in [34], Ostad-Sharif et al. in [35], Khan et al. in [38], Xu et al. in [40] and Alzahrani et al. in [41].

[SP1] Mutual authentication: Authentication is performed between SN and HN mutually in the proposed protocol. Authentication is related to the messages from SN to HN and vice versa. SN needs to be authenticated by HN based on {*X_SN_*, *Y_SN_*, *RID_S_*, *T*1*_SN_*, *PID_AP_*}, which is a message from SN to HN via AP. Only the legal SN could be authenticated by HN in the proposed protocol because a CK adversary needs to compute *RID_S_* = *h*(*ID_SN_*||*X_SN_*||*Y_SN_*||*S*2*_SN_*||*T*1*_SN_*), which needs knowledge on *ID_SN_* and *S*2*_SN_* at the same time even if the adversary could get and use the previous session’s *X_SN_* and *Y_SN_*. However, there is no way that the adversary could get them in the proposed protocol. HN needs to be authenticated by SN based on {*r*, *NX_SN_*, *NY_SN_*, *C_SN_*, *T*2*_HN_*}, which is a message from HN to SN via AP. Adversaries need to form a message, which could be validated by SN, especially *C_SN_* validation that is related with knowledge of *q*, *ID_SN_*, *j*, *X_SN_*′, *Y_SN_*′ and *T*2*_HN_*. However, the knowledge is related with *KS_HN_*, which is the master key of HN. It means that the proposed protocol provides mutual authentication between SN and HN and there is no way that the adversary could succeed in the authentication process.

[SP2] Session key agreement: Session key is required to establish a secure channel between SN and HN to provide confidentiality on data. SN and HN agree on a session key *Ks* = *h*(*q*||*S*1*_SN_*||*S*2*_SN_*) after the successful authentication. There is no way that a CK adversary could get any information on *Ks* from the session messages {*X_SN_*, *Y_SN_*, *RID_S_*, *T*1_SN_}, {*X_SN_*, *Y_SN_*, *RID_S_*, *T*1*_SN_*, *PID_AP_*}, {*r*, *NX_SN_*, *NY_SN_*, *C_SN_*, *T*2*_HN_*, *NPID_AP_*, *Z_AP_*} and {*r*, *NX_SN_*, *NY_SN_*, *C_SN_*, *T*2*_HN_*}. The parameters of *Ks* are not exposed to any parameter in the messages. Especially, *q* is related to *r* = *q* ⊕ *j* but the adversary needs to know *j* to extract out the wanted value from *r*. However, the adversary could not get *q* from *r* due to the format of *j* = *ID_SN_* ⊕ *Y_SN_* ⊕ *X_SN_*, which is related with the knowledge of *KS_HN_*. Thereby, the proposed protocol provides a secure session key agreement only between SN and HN.

[SP3] Message freshness: There are two ways to provide message freshness in cryptographic protocol, which are based on challenge-response mechanism and timestamp mechanism. The proposed protocol uses a timestamp mechanism to cope with replay attacks because the network entity could be synchronized with a time when SA issues SN and AP for a PT during the registration phase. If a CK adversary wants to succeed in any attack against message freshness, the adversary needs to know and change timestamp-related values. From the session messages {*X_SN_*, *Y_SN_*, *RID_S_*, *T*1_SN_}, {*X_SN_*, *Y_SN_*, *RID_S_*, *T*1*_SN_*, *PID_AP_*}, {*r*, *NX_SN_*, *NY_SN_*, *C_SN_*, *T*2*_HN_*, *NPID_AP_*, *Z_AP_*} and {*r*, *NX_SN_*, *NY_SN_*, *C_SN_*, *T*2*_HN_*}, there are two integrity values *RID_S_* = *h*(*ID_SN_*||*X_SN_*||*Y_SN_*||*S*2*_SN_*||*T*1*_SN_*) and *C_SN_* = *h*(*q*||*ID_SN_*||*j*||*X_SN_*′||*Y_SN_*′||*T*2*_HN_*) that the adversary needs to compute. If the adversary gets a proper current timestamp *T*1*_SN_*′, the adversary should compute two new values of *RID_S_* = *h*(*ID_SN_*||*X_SN_*||*Y_SN_*||*S*2*_SN_*||*T*1*_SN_*′) and *C_SN_* = *h*(*q*||*ID_SN_*||*j*||*X_SN_*′||*Y_SN_*′||*T*1*_SN_*′). However, the two computations are impossible because the adversary needs to know the other parameters except *T*1*_SN_*′ to compute *RID_S_* and *C_SN_*. Furthermore, each entity checks the freshness of the message using ∆*t* each time they receive any message. So, the proposed protocol provides message freshness.

[SP4] Perfect forward secrecy: It is a very strong form of long-term security which guarantees that future disclosures of some long-term secret keys do not compromise past session keys [49]. It is widely accepted that the perfect forward secrecy can only be provided by asymmetric schemes. Nonetheless, there are a small number of existing symmetric-key protocols that provide secrecy [50,51,52]. The proposed protocol uses the dynamic authentication credential, which keeps evolving in sessions to achieve the perfect forward secrecy. In the proposed protocol, if an adversary has obtained the long-term key, *K_HN_*, the adversary still cannot get the session key *K_S_*. The reason is that after each successful session, the values *HC_i_*, *S*1*_SN_* and *S*2*_SN_* will be updated by one-way hash function. Because of the one-wayness of the hash function, there is no way to get these values to compute the session key to the adversary. Therefore, the proposed protocol can provide perfect forward secrecy.

[SP5] Attack resistance: We could argue that any attack is successful if a CK adversary finds any mechanism to do various attacks, such as replay attack, impersonation attack and man-in-the-middle attack. Most of all, replay attack is tightly related with the message freshness. It means that any protocol with challenge-response or timestamp mechanism could cope with the attack. Messages in the proposed protocol are together with timestamp as the form of *T*1*_SN_* and *T*2*_HN_*, respectively. Thereby, the proposed protocol is strong against replay attack. Impersonation attack is the second one we need to consider, which has a relationship with mutual authentication. As we mentioned in the mutual authentication, the adversary needs to form the first message {*X_SN_*, *Y_SN_*, *RID_S_*, *T*1*_SN_*} to disguise as SN and the third message {*r*, *NX_SN_*, *NY_SN_*, *C_SN_*, *T*2*_HN_*, *NPID_AP_*, *Z_AP_*} to masquerade as HN, respectively. However, they are related to the knowledge of *KS_HN_*. So, the proposed protocol could cope with impersonation attacks. Man-in-the-middle attack is similar to an active eavesdropping in which the adversary makes independent connections with the network entities and relays messages between them to make them believe they are communicating directly to each other but in fact, the entire communication is controlled by the adversary. It is quite related to mutual authentication and confidentiality of parameters in the messages. Since we mentioned the mutual authentication provision from the proposed protocol, we will only consider confidentiality of the messages. There are only possibilities on knowing secret key-related information to legally registered SNs and HN but not any others. In the CK model, it is required that the generated session key from the protocol should not be compromised even in the case of ephemeral secrets leakage. In the proposed protocol, the ephemeral secrets are *a_SN_* and *q*. Having access to these two, the adversary also needs to know both *S*1*_SN_* and *S*2*_SN_* to compute the session key *K_S_*. Since only SN and HN know the values, the proposed protocol can withstand this attack. That is why any adversary could not get any useful information even if the adversary could tap into the communication link among SN, AP and HN. Thereby, the proposed protocol provides attack resilience. Finally, known session-specific temporary information attack should be considered in the protocol, which has an assumption that an adversary could get the ephemeral random number *q* to get the session key *K_S_* since the attacker has no way to compute the long-term key *KS_HN_* and one-time hash chain value *HC_i_*. Moreover, the messages transmitted in the public channel are unhelpful to compute the session key *K_S_*. Therefore, the proposed protocol has the ability to prevent the session-specific temporary information attack.

[PP1] Anonymity: Anonymity is defined as “the state of being not identifiable within a system.” Anonymity from a CK adversary’s perspective means that the adversary cannot identify any entity within a system. In security protocol, it is necessary to check identity-related information in messages transmitted among system entities to consider anonymity. There are *Y_SN_*, *RID_S_*, *NY_SN_* and *C_SN_*, for *ID_SN_* and *PID_AP_*, *NPID_AP_* and *Z_AP_* for *ID_SN_*, respectively, in the messages, which has a relationship with the identity factor. Adversaries do not have any method to identify any entity from the parameters in the proposed protocol. To do so, the adversary needs to have knowledge of *KS_HN_*, which is not feasible. As a result, the proposed protocol provides anonymity.

[PP2] Unlinkability: It has a meaning after a system with anonymity has been defined and the entities interested in linking by a CK adversary have been characterized. Unlinkability of two or more sessions of interest from the adversary’s perspective means that within the system, the adversary cannot distinguish whether they are related or not. As we discussed on anonymity, session linkability is related to the identifier and the message freshness of session message parameters. Each parameter in the session messages has a relationship with the session-dependent random numbers of *a_SN_*, *S*1*_SN_*, *S*2*_SN_*, *q* and *na_SN_* and timestamps of *T*1*_SN_* and *T*2*_HN_* in the proposed protocol. It means that the proposed protocol uses session-dependent parameters to form messages to cope with unlinkability. So, the proposed protocol provides unlinkability.

As shown in Table 2, the proposed protocol satisfies all the security and privacy properties as we set our protocol design goal in Section 2.3. However, Khatoon et al.’s protocol does not provide SP5, especially against the known-session-specific temporary information attack as mentioned in [53]. Thereby, the adversary could compute the session key *SK* in Khatoon et al.’s protocol based on the session-specific temporary information, *T_i_*, *R_i_*, *T_s_* and *R_s_*, which are parameters to compute *SK* and are exposed on the public communication channel. As stated above, the attacker can compute *L_s_*. Ostad-Sharif et al.’s protocol is weak against the denial-of-service attack, the password guessing attack and the stolen verifier attack [54]. So, Ostad-Sharif et al.’s protocol does not provide SP5 also. Furthermore, Khan et al.’s protocol has security weakness against the user impersonation attack, which is related to SP5 again [55]. Xu et al.’s protocol does not provide the replay attack since an attacker could configure a valid request by merging two session parameters by intercepting contents of the previous session and the current session parameters [41]. Alzahrani et al.’s protocol has a security weakness against the known-session-specific temporary information attack because it does not provide SP4 also. Furthermore, Xu et al.’s protocol and Alzahrani et al.’s protocol do not provide PP2 especially. In addition to this, Xu et al.’s protocol is not secure against the replay attack and the impersonation attack and does not provide PP1 due to the offline identity guessing attack feasibility [41].

## 5. Performance Results

In this section, we provide performance analysis focused on computation and communication overheads by providing comparisons with the related protocols in [34,35,38,40,41]. A dataset is developed to produce further testing and enhancements instead of spending a considerable amount of time, money and effort for data collection. 10 users were tested in the proposed protocol run for a total of 10 times. The experiment of the protocols was performed over ARM Microcontrollers MCU Mainstream Arm Cortex-M4 running on MCU 170 MHz with 128 KB of flash memory.

### 5.1. Computation Result

There are four phases in the proposed protocol, which are initialization phase, registration phase, authentication phase and identity modification phase. We will concentrate on the computation requirements of the authentication phase only from the proposed protocol because the phase is the most frequently used one. To facilitate computation analysis, we define the computational requirements of a one-way hash function as *T_h_*, a symmetric key encryption and decryption as *T_sym_*, an elliptic curve cryptosystem as *T_ecc_* and a bilinear pairing operation as *T_bp_*, respectively, but do not consider the overhead of the exclusive-or operations, which require a comparatively quite low overhead than any other operations. Table 3 shows the computational overhead comparison among the related protocols.

**Figure 5:**
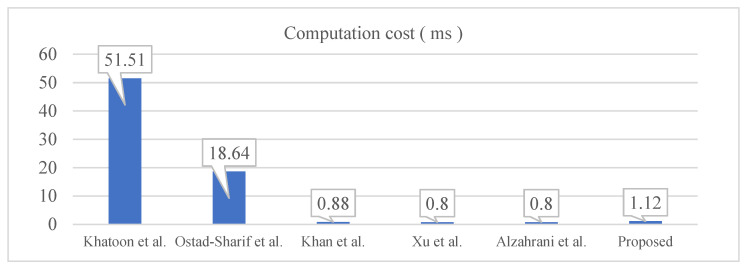
Computation cost comparison.

From the experiment, we acquired the required time for *T_h_*, *T_sym_*, *T_ecc_* and *T_bp_*, which are approximately 0.08 ms, 0.14 ms, 4.31 ms and 14.48 ms, respectively. The proposed protocol requires 14 hash operations, which is a bit more expensive than the protocols in [38,40,41] but quite lower than the works in [34,35]. However, the protocols in [40,41] do not provide the privacy concerns as we discussed in Table 2. So, we could say that the computational overhead in the proposed protocol is for the sake of privacy-preserving. Especially, it is better to get less computational overhead on the patient side than the server side as the proposed protocol. However, Khan et al.’s protocol is opposite from the notion, which has a more burden to the patient’s side. Figure 5 shows the performance comparisons among the related protocols.

From Figure 5, we could know that the proposed protocol requires about 40% more computational overhead than the protocols in [38,40,41], which could be the overhead to provide unlinkability. However, the proposed protocol is relatively lightweight compared to the protocols in [34,35].

### 5.2. Communication Result

For the communication analysis, we assumed that the lengths of identity and random numbers are 128 bits each. However, we considered that the lengths for timestamp, hash function, symmetric key cryptosystem, elliptic curve cryptosystem and bilinear pairing are 32 bits, 160 bits, 128 bits, 256 bits and 256 bits, respectively. Table 4 shows a comparison for the communication cost among the related protocols.

**Figure 6:**
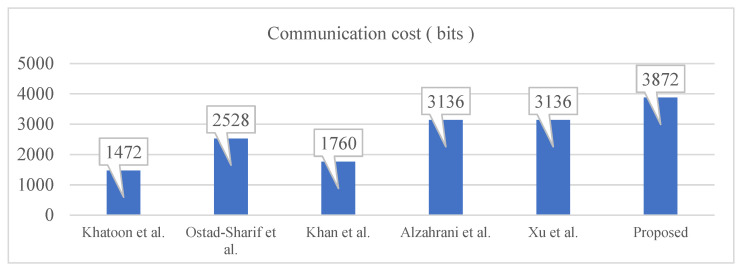
Communication cost comparison.

Protocols of Khatoon et al., Ostad-Sharif et al. and Khan et al. require 2 messages with 1472 bits, 2528 bits and 1760 bits, respectively. However, protocols of Xu et al., Alzahrani et al. and the proposed one need 4 messages of 3136 bits, 3136 bits and 3872 bits, respectively. The first three protocols in Table 4 do not involve any intermediate entity between two end parties for the communication. That is why the communication requirements are less than those four other protocols. In addition to this, the proposed protocol requires about 700 bits more than Xu et al.’s protocol and Alzahrani et al.’s protocol due to the session-dependent dynamic identifier distribution to entities in the system. As shown in Figure 6, in contrast with the computational overhead, the proposed protocol requires the heaviest communicational overhead due to the usage of AP in between SN and HN, which is different from the other protocols.

## 6. Discussion

This section discusses challenges and solutions on the authentication protocol for WBAN based healthcare applications. After that, we will provide some future work.

### 6.1. Challenges and Soluitons

Healthcare systems can provide an opportunity to meet the needs of individuals or households facing health difficulties. However, the healthcare system has an obligation to protect the privacy of patients [56]. And all participants in healthcare such as professionals of medical industries, always must be provide privacy with health data. Furthermore, healthcare professionals and medical industries around the globe are urged to fight against various security and privacy attacks on the healthcare system. WBAN based healthcare application shares some common functionalities with a typical computer network as it is a special type of network and also exhibits several unique characteristics that are specific to it. WBAN based healthcare application requires to guarantee security, privacy, data integrity and confidentiality of patient’s EHR at all times. Towards the design of efficient cryptographic solution, there are more challenges in the WBANs than wired networks. They are the wireless nature of communication, resource inadequacy on SNs and very large and dense networks. Authentication is considered as the basic security building block for any systems, which is a process by which the identity of a node in a network is verified and guarantees that the data or the control messages originate from an authenticated source. So, we will address some challenges and solutions for the authentication protocol.

The first challenge is to provide security in healthcare services that use the public network. Authentication protocol based on the public network is vulnerable against various attacks such as replay attack, impersonation attack and man-in-the-middle attack. The security issues could be overcome by utilizing various cryptographic primitives including asymmetric key cryptography, symmetric key cryptography, hash function and so on. Recently, researchers have been developing lightweight protocols, such as hash-based protocol and symmetric key cryptography-based protocol, to achieve feasibility on WBANs. Furthermore, designing authentication protocols with PUFs could help to resolve the security issues.

The second challenge is to preserve the privacy of network entities. Patient personal information is one of the most sensitive data in message transmission over the public network. The privacy issues could be dealt with by utilizing session-dependent information such as a one-time pseudonym for only the session usage. Recently, researchers have been deploying unidirectional hash chain values. A hash value from the chain is used only once and authentication protocol based on the value could provide unlinkability between sessions. In addition, cryptographic researchers should collaborate with healthcare professionals and medical industry workers to adopt and recognize various target field requirements from different backgrounds and aspects.

### 6.2. Future Work

In short, the proposed authentication protocol tries to generalize the process of mutual authentication and session key agreement for WBANs in healthcare applications. The proposed protocol takes full lightweight advantage of one-way hash function and exclusive-or operation to establish better security and privacy in solving authentication and session key establishment issues. In our future work, we aim to implement the proposed protocol in a real hospital environment with a big EHR database. We will focus on conducting experiments by optimizing patient side operational and communicational overhead of the proposed protocol to achieve better WBAN feasibility in terms of improved security and privacy. In addition, we will deploy a real-time adaptive artificial intelligence model on categorizing and analyzing EHR data to provide much richer patient healthcare services. Artificial intelligence can bring numerous benefits to the evolving of the healthcare industry. Based on artificial intelligence software, certain symptoms can be detected before the obvious symptoms of diseases such as lung cancer appear [57]. In addition, in the case of learned artificial intelligence, it can reduce the possibility of a doctor’s misdiagnosis, to reducing patient anxiety [58]. Moreover, this research work will motivate researchers to pay more attention to security and privacy and explore the combination of other technologies, such as multimedia, robots and smart cities, to provide more convenient healthcare services to patients.

## 7. Conclusions

In this paper, we proposed a privacy-preserving authentication protocol for WBANs in healthcare applications. First of all, we set our design goals focused on 5 security properties and 2 privacy requirements, which are mutual authentication, session key agreement, message freshness, perfect forward secrecy, attack resistance, anonymity and unlinkability. To satisfy those features, we designed a new authentication protocol based on only two simple and lightweight operations, hash and exclusive-or. Especially, to provide 2 privacy requirements, the proposed protocol uses session-dependent pseudo identifiers for SN and AP. The formal and informal privacy and security analyses demonstrate the resistance of the proposed protocol against all sorts of privacy and security attacks. Especially, the privacy and security features of the proposed protocol are formally verified and validated based on BAN logic and ProVerif simulation tool. Performance analysis showed that the proposed protocol has a reasonable overhead compared to the related previous protocols but still lightweight. We need to note that privacy-preserving is an important feature in healthcare service because healthcare information is sensitive. Nobody wants to expose their EHR-related information to others.

## Figures and Tables

**Table 1:** Notations.

Notation	Descriptions
*SA*	System administrator
*HN*	Healthcare central server
*PT*	Patient
*SN*	Sensor node
*AP*	Access point
*ID_SN_*	Identity of SN
*ID_AP_*	Identity of AP
*Y_SN_*	Pseudonym identity of SN
*PID_AP_*	Pseudonym identity of AP
*KS_HN_*	Long-term master key of HN
*K_S_*	Established session key
*Ti_j_*	*i*-th timestamp of an entity *j*
*Si_j_*	*i*-th random number of an entity *j*
*a_SN_, na_SN_, q*	Random numbers
*HC_i_*	Hash chain value of SN
*h*()	Secure one-way hash function
||	Concatenation operation
⊕	Exclusive-or operation
∆*t*	Allowed transmission delay

**Table 2:** Privacy and security feature comparison result.

	Protocol	Khatoon et al. [34]	Ostad-Sharif et al. [35]	Khan et al. [38]	Xu et al. [40]	Alzahrani et al. [41]	Proposed
Feature							
SP1		O	O	O	O	O	O
SP2		O	O	O	O	O	O
SP3		O	O	O	O	O	O
SP4		O	O	X	X	X	O
SP5		X	X	X	X	X	O
PP1		O	O	O	X	O	O
PP2		O	O	O	X	X	O

SP1: mutual authentication, SP2: session key agreement, SP3: message freshness, SP4: perfect forward secrecy, SP5: attack resistance, PP1: anonymity, PP2: unlinkability.

**Table 3:** Computation cost comparison result.

	Protocol	Khatoon et al. [34]	Ostad-Sharif et al. [35]	Khan et al. [38]	Xu et al. [40]	Alzahrani et al. [41]	Proposed
Entity							
SN		5*T_h_* + 1*T_bp_* + 1*T_sym_* + 3*T_ecc_*	7*T_h_* + 2*T_ecc_*	7*T_h_*	4*T_h_*	4*T_h_*	4*T_h_*
AP		-	-	-	-	-	1*T_h_*
HN		*4T_h_* + 1*T_bp_* + 1*T_sym_* + 2*T_ecc_*	7*T_h_* + 2*T_sym_* + 2*T_ecc_*	4*T_h_*	6*T_h_*	6*T_h_*	9*T_h_*
Total		9*T_h_* + 2*T_bp_* + 2*T_sym_* + 5*T_ecc_*	14*T_h_* + 2*T_sym_* + 4*T_ecc_*	11*T_h_*	10*T_h_*	10*T_h_*	14*T_h_*

**Table 4:** Communication cost comparison result.

	Protocol	Khatoon et al. [34]	Ostad-Sharif et al. [35]	Khan et al. [38]	Xu et al. [40]	Alzahrani et al. [41]	Proposed
Feature							
Message length	SN	832 bits	1408 bits	1120 bits	896 bits	896 bits	896 bits
	AP	-	-	-	1024 bits + 544 bits	1024 bits + 544 bits	1312 bits + 480 bits
	HN	640 bits	1120 bits	640 bits	672 bits	672 bits	1184 bits
	Total	1472 bits	2528 bits	1760 bits	3136 bits	3136 bits	3872 bits
Number of messages		2 messages	2 messages	2 messages	4 messages	4 messages	4 messages

## Data Availability

Data could be downloaded with the following URL at https://github.com/hs-kim-andre/healthcare.git, accessed on 26 August 2021.
